# Genetic background influences pig responses to porcine reproductive and respiratory syndrome virus

**DOI:** 10.3389/fvets.2023.1289570

**Published:** 2023-10-20

**Authors:** Yangli Pei, Chenghong Lin, Hua Li, Zheng Feng

**Affiliations:** Guangdong Provincial Key Laboratory of Animal Molecular Design and Precise Breeding, Key Laboratory of Animal Molecular Design and Precise Breeding of Guangdong Higher Education Institutes, School of Life Science and Engineering, Foshan University, Foshan, China

**Keywords:** porcine reproductive and respiratory syndrome, pig breeds, genetic backgrounds, PRRSV, PRRSV receptors, innate immunity, acquired immunity

## Abstract

Porcine reproductive and respiratory syndrome virus (PRRSV) is a highly infectious and economically significant virus that causes respiratory and reproductive diseases in pigs. It results in reduced productivity and increased mortality in pigs, causing substantial economic losses in the industry. Understanding the factors affecting pig responses to PRRSV is crucial to develop effective control strategies. Genetic background has emerged as a significant determinant of susceptibility and resistance to PRRSV in pigs. This review provides an overview of the basic infection process of PRRSV in pigs, associated symptoms, underlying immune mechanisms, and roles of noncoding RNA and alternative splicing in PRRSV infection. Moreover, it emphasized breed-specific variations in these aspects that may have implications for individual treatment options.

## 1. Introduction

Porcine reproductive and respiratory syndrome (PRRS) is a highly destructive disease that was first identified in the United States in 1987, and later spread to Europe in 1990 (1, 2). It poses a considerable economic risk to the swine industry (3–5). The estimated economic impact of PRRS on the entire herd of four Chinese farms experiencing outbreaks is ¥1424.37 per sow (4). The PRRS virus (PRRSV) is the pathogen responsible for causing PRRS, characterized by its positive-stranded RNA nature and enveloped structure (6). It belongs to the order Nidovirales and the family *Arteriviridae* (6). PRRSVs are classified into PRRSV-1 and -2 genotypes that occur in Europe and North America, respectively (7). PRRSV-1 and -2 share approximately 60% nucleotide identity (1, 8); however, it is believed that they underwent separate evolutionary paths, originating from a distant common ancestor (9). PRRSV-2 primarily targets cells of the monocyte/macrophage lineage, particularly porcine alveolar macrophages (PAMs) (10).

The Prevention and control of PRRS poses a significant challenge. Current strategies include vaccination, herd management, biosecurity, and antiviral treatment (11). However, the effectiveness of these measures varies depending on specific circumstances and implementation strategies. Vaccination shows promising results in reducing the incidence and severity of PRRS; however, it does not offer a complete solution (12). Effective herd management (including monitoring and controlling pig movement) can help reduce the disease spread. Furthermore, biosecurity measures, such as disinfection and cleaning of facilities may help to prevent disease transmission (11). Although antiviral treatment can reduce disease severity, although it is not a permanent cure and may not be cost-effective in all situations (13). Therefore, further research is necessary to identify and develop more effective methods to prevent and control PRRS.

The genetic background of pigs is a significant factor determining their response to PRRSV. Various pig breeds and lines exhibit different levels of resistance to PRRSV infection (14, 15). Meishan and Tongcheng (TC) breeds, known for their elevated resistance to PRRSV, are less susceptible to infection when compared to other breeds, such as the Large White (LW) (16–18). Moreover, specific pig tissues such as the lungs and lymph nodes may exhibit varying susceptibilities to PRRSV, which may be influenced by genetic factors (19). Enhanced knowledge of the genetic factors contributing to the differences in PRRSV resistance among pig breeds potentially improves pig health and welfare, ultimately reducing the economic losses associated with PRRSV infection.

Overall, controlling PRRS remains a challenge, owing to the complex host-pathogen interactions despite extensive research efforts. Further research is required to develop effective countermeasures against the virus. This review focuses on the influence of genetic factors on pig responses to PRRSV, including differences among pig breeds and lines. Furthermore, we investigated PRRSV infection mechanisms and factors affecting the host response, such as the innate and adaptive immune systems. Additionally, alternative splicing events and noncoding RNAs involved in PRRSV infection and replication were explored. The potential implications of this research were to develop effective control strategies and breeding programs that utilize genetic information to improve pig health and productivity. This extensive knowledge will potentially enhance pig well-being, increase productivity, and promote worldwide sustainability of pig farming.

## 2. Varied receptor responses in different pig breeds upon PRRSV invasion

### 2.1. Mechanisms of host cell entry

The PRRSV genome is approximately 15 kbp in size and has a specific organization. The replicase genes are situated at the 5′-end of the genome, whereas the genes encoding structural proteins are found at the 3′-end (20, 21). The viral genome consists of more than 10 open reading frames (ORFs). Over 66% of the viral genome is made up of ORF1a and ORF1b, which encode nonstructural proteins that serve crucial functions including protease and replicase activities. These proteins also modulate host genes that are vital for the replication of the virus. Conversely, ORFs 2–7 encode the structural proteins required for virus formation (21).

PRRSV processes of eight structural proteins, which include a small non-glycosylated protein and a group of glycosylated proteins: Glycoprotein (GP) 2ab, GP3, GP4, GP5, GP5a, matrix (M), and nucleocapsid (N) (21, 22). The primary structural proteins encoded by ORFs 5, 6, and 7 are GP5, M, and N, respectively. While GP5 typically forms a heterodimer with M, there have been reports of GP5 homodimers (23). Among the surface glycoproteins, GP2, GP3, and GP4, derived from ORFs 2, 3, and 4, respectively, act as minor components. Additionally, two very small non-glycosylated proteins, designated as 2b or E and 5a, are translated from ORF2b and ORF5a, respectively (24, 25). The smooth exterior of the PRRSV virion is primarily attributed to the presence of short peptide sequences in the ectodomains of M and GP5. However, the larger ectodomains of GP2, GP3, and GP4 can also give rise to a few protrusions on the virus surface (21).

Macrophages are the primary target cells for PRRSV infection (10, 26–28), playing a crucial role in immune modulation and contributing to the respiratory distress observed in pigs affected by the porcine respiratory disease complex. The following section provides an overview of the recognition of the virus by recipient cells (Table 1) and the mechanisms involved.

**Table 1:** Major receptors and function during PRRSV infection.

Receptor	Function	References
CD163	CD163 interacts with PRRSV glycoproteins GP4 and GP2a to form a multiprotein complex. The presence of CD163 SRCR5 is essential for PRRSV infection, with its absence in pigs conferring resistance against different PRRSV strains. Furthermore, CD163 plays a vital role in TIM-induced macropinocytosis during PRRSV infection	(29–36)
Heparin sulfate (HS)	HS interacts with the M and GP5-M proteins of PRRSV, enabling efficient attachment of the virus to cells. However, it is not a prerequisite for PRRSV invasion of macrophages	(37, 38)
Sialoadhesin (Sn)	The N-terminal immunoglobulin domain of porcine Sn is critical factor that promotes PRRSV attachment to porcine alveolar macrophages (PAMs). Although it does not contribute to viral uncoating, Sn facilitates PRRSV internalization in expressing cells	(39–44)
Vimentin	Vimentin directly interacts with PRRSV nucleocapsid protein, and the application of antivimentin antibodies effectively block PRRSV	(45–48)
CD151	CD151 functions as an RNA-binding protein, and directly interacts with the 3′-UTR RNA of PRRSV. CD151 overexpression enhances PRRSV infection in non-susceptible cells, whereas blocking CD151 with antibodies inhibits PRRSV infection in susceptible cells	(49, 50)
Non-muscle myosin heavy chain 9 (MYH9)	Interaction of MYH9 with PRRSV GP5 protein is indispensable to facilitate PRRSV internalization and intercellular spread	(51–53)
Heat shock protein member 8 (HSPA8)	The PB domain of HSPA8 interacts with PRRSV GP4, and the ATPase activity of HSPA8 is crucial for PRRSV infection through clathrin-mediated endocytosis (CME). Furthermore, HSPA8 co-localizes with PRRSV on the cell surface, and plays a pivotal role in facilitating viral fusion and entry	(54, 55)

CD163 serves as a crucial receptor for PRRSV and plays a critical role in determining cell susceptibility to the virus (29). It is a scavenger receptor glycoprotein predominantly found in mature macrophages and monocytes. The extracellular portion of CD163 comprises nine scavenger receptor cysteine-rich domains (SRCR) and two motifs rich in proline-serine–threonine (PST), which are repeated multiple times (56). The heterotrimeric GP2, GP3, and GP4 proteins of PRRSV bind to CD163, with GP2 and GP4 forming multiple interactions with different receptors (30, 56). Pigs with a *CD163* gene knockout (KO) are non-permissive to PRRSV-2 infection (31), and their macrophages show resistance to PRRSV-1 and -2 (32). Recent studies show that CD163 SRCR5-deficient pigs are resistant to specific PRRSV-2 strains (33, 34). Genetically engineered pigs with a modified CD163 SRCR5 domain display normal growth under standard conditions (34, 35). This suggests that gene editing techniques targeting CD163 can potentially control and eradicate PRRS outbreaks.

CD163 participates in viral apoptotic mimicry, a strategy employed by certain viruses to infect host cells (57). This mechanism involves viruses disguising themselves as apoptotic debris and engaging receptors on the surface of phagocytes that recognize phosphatidylserine (PtdSer), a marker of apoptosis (58). These interactions activate signaling cascades and lead to actin rearrangements to facilitate endocytic engulfment, and subsequent degradation of viral particles (59). Viruses adopt distinct mechanisms of apoptotic mimicry, giving rise to the concepts of classical and nonclassical apoptotic mimicry (60). PRRSV capitalizes on viral apoptotic mimicry to induce macropinocytosis through the involvement of T cell immunoglobulin and mucin domain proteins TIM-1 and -4 (57). During PRRSV infection, CD163 plays a vital role in facilitating TIM-induced macropinocytosis (61). Consequently, PRRSV adopts an alternative route of infection via macrophage activity involving CD163.

Mammalian cells contain heparin sulfate (HS) as a glycosaminoglycan on their surface and in their extracellular matrix (62). Heparin sulfate plays an important role in adhesion during PRRSV infection by interacting with M and GP5-M proteins during PRRSV infection (37). Although not essential for PRRSV invasion of porcine alveolar macrophages (PAMs), HS enables PRRSV to adhere to non-susceptible cell lines without completing the subsequent infection steps (38). PRRSV-1 and -2 exhibit different sensitivities to HS (37). The treatment of PAMs with heparinase (which degrades HS) reduces PRRSV infection (37). Moreover, PRRSV infection can activate NF-κB and cathepsin L, resulting in heparinase upregulation and processing, reduction in HS surface expression, and promotion of viral replication and release (63).

Sialoadhesin (Sn), also referred to as CD169 or SIGLIC-1, acts as a co-receptor for PRRSV invasion. The N-terminal immunoglobulin domain of porcine Sn is necessary and sufficient (39, 40). Cells expressing Sn facilitate PRRSV internalization, but do not promote viral uncoating (40). The collaboration between Sn and other receptors, such as CD163, sensitizes cells to PRRSV infection, promoting effective attachment, internalization, and disassembly of viral particles (41, 42). The absence of Sn in genetically-edited pigs does not disrupt PRRSV attachment/internalization or have any effect on disease progression or histopathology (64). This discrepancy between the *in vitro* and *in vivo* models of PRRSV receptors indicates conflicting outcomes. These findings suggest that Sn primarily plays a role in binding PRRSV to the macrophage surface rather than facilitating viral internalization.

Vimentin (VIM) is a crucial component of the PRRSV receptor complex and plays a key role in the intracellular replication and metastasis of PRRSV (45, 46). It forms a polymer with other fine-cell bone frame microfilaments and interacts with the PRRSV nucleocapsid protein (47). Vimentin can render normally non-susceptible cell lines susceptible to PRRSV infection. This highlights its involvement in the viral receptor complex (45). Following PRRSV entry, vimentin undergoes reorganization facilitated by Serine 38 phosphorylation by calcium calmodulin-dependent protein kinase II gamma (48). As a result of this reorganization, cage-like structures form around PRRSV replication complexes within the nucleus.

Tetraspanin superfamily member CD151 is an RNA-binding protein that interacts with the 3’-UTR of the PRRSV genome and functions as an RNA-binding protein (49). By silencing the CD151 gene in MARC-145 cells, PRRSV infection decreases significantly, and antibodies against CD151 prevent it entirely (49). CD151 expression can be regulated by microRNAs (such as miR-506) that lead to a decrease in CD151 mRNA and protein levels, thereby resulting in the inhibition of PRRSV replication and viral release in MARC-145 cells (50). CD151 possesses N-glycosylation and palmitoylation sites (65); their involvement in regulating PRRSV infection requires further investigation.

Non-muscle myosin heavy chain 9 (MYH9) plays various roles in cell adhesion, polarization, morphogenesis, and migration (66, 67). It interacts with PRRSV GP5, which is crucial for PRRSV internalization and intercellular spread. The C-terminal domain of MYH9 directly binds to the first ectodomain of viral GP5, and disruption of this interaction reduces PRRSV internalization (51). Specific amino acid residues within the MYH9 C-terminal domain are believed to be key binding sites for GP5 (52). Furthermore, MYH9 undergoes reorganization upon PRRSV infection, forming cage-like structures around the PRRSV replication complex (53). MYH9 co-expression with CD163 enhances PRRSV infection (51).

Heat shock protein member 8 (HSPA8) plays a role in various viral infections by regulating viral entry, replication, and assembly (68). Inhibition of endogenous HSPA8 reduces PRRSV replication by decreasing viral attachment and internalization (54). HSPA8 interacts with clathrin, and is involved in clathrin-mediated endocytosis (CME) (69). The PB domain of HSPA8 interacts with PRRSV GP4, and HSPA8 ATPase activity is required for PRRSV infection via CME (54). Moreover, HSPA8 co-localizes with PRRSV on the cell surface and facilitates viral fusion and entry (54). HSPA8-based therapies show promise in clinical trials and vaccine development for other diseases. For instance, recombinant HSPA8 fused with GP3 and GP4 boosts immune responses and confers protective effects against the highly pathogenic PRRSV infection in pigs (55). Therefore, HSPA8-based strategies have the potential for vaccine development.

The viral genome contains more than 10 ORFs. These ORFs encode nonstructural proteins crucial for viral replication. ORFs 2–7 encode structural proteins that play vital roles in viral formation, and are necessary for viral particle assembly. PRRSV uses multiple receptors for entry into host cells, including CD163, Sn, HS, VIM, MYH9, CD151, CD209, and HSPA8. Understanding the interactions between PRRSV and these receptors offers potential targets to control and eradicate PRRSV outbreaks, and to develop vaccines and therapeutic strategies.

### 2.2. Different pig breeds show varying expression of PRRSV receptor genes in lung tissues upon PRRSV infection

Chinese Dapulian pigs (DPL) exhibit increased resistance to PRRSV when compared to commercial Duroc×Landrace×Yorkshire (DLY) crossbred pigs, as evidenced by lower rectal temperatures and serum PRRSV copy numbers (70). Analysis of lung tissue samples from PRRSV-uninfected DPL and DLY pigs show varied expression patterns of five PRRSV mediator genes (NMMHC-IIA, SIGLEC1, CD163, HSPG2, and VIM), with significantly higher mRNA expression levels of SIGLEC1, NMMHC-IIA, CD163, and VIM in DLY pigs than those in DPL pigs (70). Another study revealed that the mRNA level of CD163 in PAMs of Dingyuan pigs is significantly lower than that of Jiangquhai pigs within 24 h post-infection (hpi); this may account for the high resistance of Jiangquhai pigs to PRRSV (71). Moreover, Sn expression in PAMs from Dingyuan pigs increase at a faster rate than that in PAMs from Jiangquhai pigs following viral infection (71). This finding corresponds to the high PRRSV content in PAMs from Dingyuan pigs (71). The increased mRNA expression levels of CD163 and Sn may contribute to more rapid viral invasion and wider penetration sites *in vivo* and *in vitro* (72). Viral receptor expression varies among different pig breeds after PRRSV infection, and there are variations in the expression of PRRSV receptors (HS, Sn, CD163, CD151, and VIM) in the lung tissues of different pig breeds, even under normal physiological conditions (47, 71, 73).

Variations in receptor expression and RNA abundance can lead to activation or inhibition of various pathways, resulting in distinct responses to PRRSV infection in different pig breeds. Further exploration of the mechanisms underlying these susceptibility differences could pave the way for the development of innovative approaches to control PRRSV infection in swine populations.

## 3. Variation in manifestations in different pig breeds during PRRSV-infection

### 3.1. Symptoms and lesions after PRRSV infection

The clinical manifestations of PRRS are influenced by multiple factors, including the virus strain, age and immune status of the host, production environment, productive state, and specific PRRSV strains. The typical symptoms during the acute phase of the disease include loss of appetite, weakness, fever, and respiratory difficulties. Respiratory dyspnea is commonly observed across all the age groups of pigs, although infected pregnant sows may exhibit more severe symptoms.

Pregnant sows are highly susceptible to PRRSV infection and may exhibit various clinical symptoms. This includes loss of appetite, abortions, transient discoloration of the ears (commonly known as blue ear disease, which affects approximately 2% of sows), early farrowing, prolonged anestrus, delayed return to heat after weaning, coughing, and respiratory signs (74).

Symptoms in weaned and fattened piglets can be significant and may include hair loss, slight loss of appetite, mild respiratory problems (such as coughing), and localized skin redness. The mortality rate during this stage ranges from 10 to 20% and is influenced by hygiene and operational management. The presence of other microorganisms within a herd can increase mortality rates. Pigs aged 4–12 weeks born by infected sows exhibit clinical symptoms similar to those of suckling pigs, including loss of appetite, malabsorption, wasting, coughing, and pneumonia, and a 12% higher post-weaning mortality rate (75, 76). Secondary bacterial infections can lead to lung and systemic abscesses, abscess-related lameness, or poor growth (77, 78).

A range of clinical symptoms indicates potential issues in farrowing sows. These symptoms include anorexia, decreased water intake, reduced milk production, mastitis, premature delivery of piglets, discoloration of the skin (such as, verticillium wilt or blue vulva and ears), pressure sores, lethargy, respiratory symptoms (such as, coughing and pneumonia), mummified piglets, stillborn piglets, and weak piglets at birth (77, 79).

Severe respiratory diseases and decreased survival rates are the most common issues in piglets. Other clinical symptoms included eyelid swelling, conjunctivitis, listlessness, significant weight loss, diarrhea, rough and unkempt fur, purple ear discoloration, and abnormal behavior (76, 77).

### 3.2. Clinical features of PRRSV infected pigs

Artificial infection of TC pigs and LW pigs with HP-PRRSV results in similar symptoms of high fever. LW pigs have a temperature above 40.5°C from 0 to 3 days post-contact (dpc) and above 41.0°C from 4 to 7 dpc, while TC pigs have a temperature above 40.5°C from 1 to 3 dpc and above 41.0°C from 4 to 6 dpc (18). However, the clinical signs are less severe in TC pigs than those in LW pigs, showing changes in lying behavior, less depression, deep breathing, skin flushing, and reduced food intake. Furthermore, TC pigs have significantly less inflammatory exudation (*p* < 0.01) and alveolar wall thickening (*p* < 0.05) when compared with LW pigs. Post-mortem analysis revealed varying degrees of swelling and bleeding in the brain, liver, and spleen, with jagged edges in the spleen (18).

Similarly, a study involving 4-6-week-old piglets of Tibetan, ZangMei black (ZM), and LW piglets challenged with HP-PRRSV (JXA1) showed that LW piglets had a significant increase in rectal temperature from 2 dpi that remained elevated until 15 dpi (40.4°C ± 0.55). ZM piglets exhibit a significant increase in rectal temperature for 4 days (2–5 dpi) with no readings above 40°C, and Tibetan piglets did not show any rectal temperature readings above 40°C. Anorexia, sneezing, coughing, and diarrhea appeared in the affected ZM and LW piglets within 2–3 dpi; however, LW piglets experienced more severe symptoms, including increased shivering, hyperspasmia, and respiratory rates from 6 to 8 dpi. Some of the challenged LW piglets died at 9, 11, and 13 dpi, whereas Tibetan piglets did not exhibit typical signs or death throughout the 28-day period (80). The observed clinical signs were aligned with corresponding changes in temperature and body weight gain. LW piglets showed decreased body weight during the second week following challenge, ZM piglets experienced weight loss during the first week, and Tibetan piglets showed consistent weight gain throughout the 4 weeks (80).

Porcine reproductive and respiratory syndrome virus infection has a notable effect on the clinical presentation and growth performance of pigs, which can significantly vary among breeds. For example, LW pigs experience weight loss and mortality after PRRSV infection, whereas ZM pigs show no weight changes. In contrast, Tibetan pigs (known for their robust disease resistance) continue to exhibit weight gain throughout the infection (80).

### 3.3. Viral load after PRRSV infection

TC pigs display significantly lower viral loads than LW pigs after artificial HP-PRRSV infection. Moreover, TC pigs display a reduced peak viral load and maintain a steady decline throughout the infection period. The maximum quantity of PRRSV particles in TC pigs is 0.4 times that found in LW pigs; this suggests a superior ability to control viral replication in TC pigs (18).

A study of 100 pigs from NEI (a Large White-Landrace composite population) and 100 pigs from a cross between Hampshire and Duroc line (HD) inoculated with PRRSV (97–7895 strain) indicated that the viremia titer was greater in HD pigs than that in NEI pigs on days 4, 7, and 14, whereas the viral titers in the lungs and bronchial lymph nodes were significantly higher in HD pigs (14).

A study involving seven Miniature (MI) pigs and eight commercial Pietrain (PI) pigs challenged with an attenuated PRRSV strain shows that viremia peaks at 6 dpi, with 100% viremia observed in PI pigs and at 12 dpi, with 87% viremia noted in MI pigs (81). MI pigs have a reduced duration of viremia. Different genetic susceptibilities to PRRSV may contribute to variations in antibody production (81).

Inoculation of eight purebred boars (two Landrace, three Yorkshire, and three Hampshire) with VR-2332 detected PRRSV RNA in the serum and PBMC between 4 and 11 dpi. The virus is not detected in the lymphoid tissues of Landrace pigs at 47 and 88 dpi, whereas Yorkshire and Hampshire pigs show varying viral loads in lymphoid tissues. Yorkshire and Hampshire boars exhibit higher resistance to PRRSV shedding in semen than Landrace boars (82).

In 2015, Tibetan, Zang Mei, and Large White piglets were challenged with HP-PRRSV (JXA1). During the challenge, the serum viral load in LW piglets peaked at 7 dpi, which gradually decreased until 28 dpi. Zang Mei piglets had their viral peak virus at 4 dpi, which decreased rapidly, resulting in significantly lower viral loads when compared to LW piglets at 14 and 21 dpi (80). Tibetan pigs consistently exhibited lower viral loads than the other two breeds throughout the challenge (80).

The virus titer and mRNA abundance in PAM supernatants following inoculation with PRRSV NJGC *in vitro* show similar trends among Landrace, Erhualian, Suzhong, Jiangquhuai, Dingyuan, and Meishan breeds (71).

Different breeds exhibit significant variations in clinical features, growth performance, and viral titers owing to genetic variations that ultimately affect their ability to combat PRRSV. Some breeds, such as Meishan and Tongcheng, demonstrate a genetic advantage in fighting the virus that results in a reduction in the duration of viremia.

## 4. Varied immune responses in different pig breeds infected by PRRSV

The innate immune response serves as the initial defense against PRRSV that is known for its ability to evade the host immune system by downregulating pattern recognition receptors (PRRs) (83). Despite viral evasion strategies, the innate immune response plays a vital role in controlling viral spread, minimizing tissue damage, and initiating the adaptive immune response (84). Essential cytokines [including interleukin-1 (IL-1), interleukin-6 (IL-6), interleukin-10 (IL-10), and tumor necrosis factor-alpha (TNF-α)] have multifaceted roles in influencing the outcome of PRRSV infection (Table 2) (16, 85–91). These cytokines modulate inflammation, exert antiviral effects, and activate immune cells (92).

**Table 2:** Summary of different virus strains affecting different cell cytokines.

Virus strain	Cell line	Cytokines	References
JX, HV, VR2332	PAM	IL6↑	(85)
CH-1a, HV	PAM	IL8↑	(86)
CH-1a	PAM	IL15↑	(87)
JXwn06, CH-1a	PAM	IL12p40↑	(88)
WuH3	MARC-145	TNF-α↑	(16)
HV, CH-1a	PAM, MARC145	IL17↑	(89)
NVSL 97-7895	moDCs	IL10↑	(90)
PRRSV-23983	moDCs	IFN-α↑	(91)

The adaptive immune system (including antigen-presenting cells) offers broader and more sophisticated recognition of antigens (93). It relies on the specific recognition of antigens by T and B cells that are generated through gene rearrangements during lymphocyte development, resulting in unique but limited specificity (93). The innate immune system is vital for eliminating viruses; however, it may not always be adequate for eradicating pathogens (93). Acquired immune functions are responsible for eliminating viruses and providing long-term immunity.

The adaptive immune response to PRRSV involves the activation of T and B cells, resulting in the production of targeted antibodies and development of cellular immune responses (94). These immune responses are crucial to eradicate the virus and protect against reinfection. The intricate mechanisms underlying the adaptive immune response to PRRSV were extensively explored in previous reviews (94, 95).

Differences in immune responses were observed among various pig breeds infected with PRRSV (14, 15, 17–19, 47, 82, 96–101). For instance, TC pigs demonstrate enhanced resistance to PRRSV with milder clinical symptoms, fewer lung lesions, and lower viremia levels when compared to other breeds, such as LW pigs. These distinctions can be attributed to genetic factors and variations in cytokine levels. TC pigs show higher serum levels of interferon-gamma (IFN-γ) that is associated with a T cell-mediated cellular immune response, whereas LW pigs show elevated levels of interleukin-10 (IL-10) that can inhibit viral clearance and impede the immune response (17, 18). These indicate the presence of genetic variations that influence viral resistance or susceptibility.

Further analysis of lymph nodes from TC and LW pigs infected with PRRSV revealed genetic differences in antigen presentation, metabolism, and immune activation; this suggests that genetic variations contribute to divergent immune responses (17). Integrated analysis of transcriptomic and metabolomic data provides additional insights into the immune response to PRRSV infection by highlighting the importance of immune activation, antigen recognition capacity, cell metabolism, and the cell cycle in the clearance of PRRSV. This analysis reveals differences in lipid metabolism and amino acid pathways between resistant and susceptible pigs, and further illustrates the impact of genetic factors on immune response (98).

A comparison of the innate immune responses of conventional and specific-pathogen-free (SPF) Yorkshire pigs to PRRSV shows that SPF pigs have elevated levels of pro-inflammatory cytokines, such as IL-1β, IL-6, and TNF-β, and higher IFN-β concentrations when compared to conventional pig breeds (102). Furthermore, SPF pigs exhibit lower viral RNA levels and less severe clinical symptoms in response to PRRSV infections (102). Meanwhile, a study of the innate immune responses of LW and Meishan pigs to PRRSV revealed that the Meishan breed produces a higher concentration of IFN-α and has lower viral loads when compared to the LW breed; this suggests a greater resistance to PRRSV owing to differences in their innate immune response (16).

Additionally, the viral genomic diversity of PRRSV (including differences in immune epitopes) contributes to its ability to evade the immune system (22). Genetic variability among PRRSV strains affects the response of pig breeds to infections. Landrace, Large White, and Yorkshire breeds are more susceptible to PRRSV, whereas Pietrain, Meishan, and Hampshire breeds are relatively resistant owing to stronger innate immune responses (82, 103). Dendritic cells derived from Pietrain pigs elicit a more potent T cell response; this underscores the significance of innate immunity in adaptive immunity (104). The susceptibility of pig breeds to PRRSV infection is primarily determined by genetic factors rather than environmental or husbandry factors.

Variations in genes associated with the innate immune response and resistance to PRRSV are identified. For example, single-nucleotide polymorphisms (SNPs) in genes such as *EIF2AK2*, *CD163*, *CD169*, and *RGS16* are linked to increased resistance against PRRSV infection (105).

The differences in innate immunity observed among various pig breeds suggest disparities in their genetic makeup. Implementing breeding initiatives aimed at enhancing innate immunity may be a valuable strategy to boost the health and productivity of swine herds. Breeders and researchers can identify the genetic markers responsible for heightened innate immunity and increased resistance to PRRSV by delving into the mechanisms that regulate innate immunity,

## 5. Differential alternative splicing events triggered by PRRSV invasion in different pig breeds

Alternative splicing (AS) is a crucial mechanism for post-transcriptional RNA processing and is responsible for significant modification of transcript sequences (106, 107). It is a critical mechanism in the regulation of eukaryotic gene expression via transcriptional control that enhances the versatility and diversity of transcriptomes and proteomes (108–111). This results in various alternative splicing events (ASEs), including skipped exons (SE), retained introns (RI), alternative 5′ and 3′ splicing sites (A5SS and A3SS), and mutually exclusive events (ME) (112, 113).

Porcine reproductive and respiratory syndrome virus infection profoundly affects alternative splicing in pigs. Specifically, immune response-related genes (such as interferon-stimulated genes) exhibit alternative splicing following PRRSV infection (114). This suggests that alternative splicing may play a role in regulating the host response to viral infections, such as PRRSV. Additionally, PRRSV infection can trigger widespread AS events in the spleen and inguinal lymph nodes (ILN) of TC and LW pigs. PRRSV infection resulted in 373 and 595 genes displaying differential ASEs in the spleen and ILN in TC pigs, respectively (114). Meanwhile, 458 and 560 genes exhibit differential ASEs in the spleen and ILN, respectively in LW pigs. Gene Ontology functional analysis revealed that these genes are important for immune responses, transcriptional regulation, metabolism, and apoptosis (114). Furthermore, the response to PRRSV in terms of alternative splicing significantly differed between the TC and LW pigs. This suggests a possible link between PRRSV infection and genetic variation in these two pig breeds.

## 6. Functions of host non-coding RNAs in PRRSV infection and replication

Non-coding RNAs (ncRNAs) play diverse roles in PRRSV infection and replication. PRRSV is an RNA virus possessing a long untranslated region (UTR) downstream of its open reading frame, 1ab (21). Research suggests that the 3’-UTR plays a significant role in modulating targeted mRNAs in animals (115, 116). This indicates that the extended UTR in PRRSV could potentially serve as a pool of targets for host miRNAs.

miRNAs play a critical role in regulating viral replication and the host immune response infection during PRRSV. For example, miR-181 inhibits PRRSV *in vivo* and *in vitro*, and therapeutic delivery of miR-181 alleviates symptoms and prolongs the survival of highly pathogenic PRRSV-infected pigs (117). Furthermore, it downregulates the PRRSV receptor CD163, effectively hindering PRRSV infection. The construction of an miR-181 target site-mutated PRRSV demonstrated that miR-181 effectively blocked wild-type PRRSV invasion in the late stage, suggesting its significant impact on PRRSV infection and replication *in vivo*. Additionally, cellular miR-23 inhibits PRRSV replication by directly targeting PRRSV RNA and potentially upregulating type I interferon (118). MiR-378 and miR-505 suppress PRRSV replication by directly targeting PRRSV RNA (118), whereas miR-10a-5p inhibits PRRSV replication by suppressing SRP14 expression (119).

In contrast, PRRSV-induced miR-142-5p significantly promotes viral replication by directly targeting FAM134B (120). Conversely, let-7 family miRNAs inhibit PRRSV replication by targeting the 3’-UTR of the PRRSV-2 genome and porcine IL-6 (121). MiR-146a expression increases in macrophages during PRRSV infection, and positively affects the immune response by regulating the expression of genes, such as *C1QTNF3* and *MAFB* (122). Notably, neither PRRSV-infected target cells nor host pigs induce the production of type I interferon (IFN) proteins *in vivo* or *in vitro* (123). MiRNAs can be induced or repressed by type I IFN, although they can also play key roles in regulating innate immune responses by modulating the production of type I IFN and other important molecular pathways (124). In particular, miR-331-3p/miR-210 is involved in lung inflammation by targeting ORF1b and downregulating STAT1/TNF-α (16).

Let-7b, miR-26a, miR-34a, and miR-145 directly target sequences within the porcine IFN-β 3 β-UTR regions at positions 160–181, 9–31, 27–47, and 12–32 bp, respectively, to inhibit the expression of IFN-β protein in primary PAMs (125). Moreover, it is suggested that PRRSV can suppress the post-transcriptional expression of IFN-β protein by upregulating these four miRNAs in cultured PAMs (126). MiR-199a-3p downregulates the protein expression of CD151, a receptor utilized by PRRSV (125). Additionally, miR-199a-3p is differentially expressed in the lung tissues of different pig breeds (such as, Tongcheng and Landrace) in the control and infection groups; this suggests its crucial role in regulating PRRSV infection in pigs (127). Furthermore, miR-378 and miR-10a-5p are upregulated in the control group of Tongcheng pigs when compared to those in the control group of Landrace pigs, and both miRNAs showed inhibitory effects on PRRSV replication (126).

Numerous studies have explored the role of miRNAs in the PRRSV process, shedding light on their regulatory mechanisms in viral infection, and revealing variations in miRNA expression among different pig breeds. These findings provide novel insights into the interaction between PRRSV and the host, and present promising avenues to develop antiviral strategies against PRRSV infection.

## 7. Future perspectives

Future research should focus on several key areas to advance our understanding of PRRSV infections and drive the development of effective control strategies.

First, extensive studies are required to investigate the genetic factors underlying the varied responses to PRRSV infection among different pig breeds. Identifying specific genes and genetic markers associated with PRRSV resistance or susceptibility will provide valuable insights into targeted breeding programs and the development of precision medicine approaches. Through genetic improvement and selective breeding methods, it is possible to rear pig breeds that exhibit enhanced resistance to PRRSV. Leveraging modern genetic engineering technologies and selective breeding methods, individuals with robust immune responses can be selected for reproduction, thereby gradually improving the overall PRRSV resistance within the entire pig population.

Second, rapid advancements in single-cell transcriptome sequencing and spatial transcriptomics present opportunities to gain in-depth molecular insights into PRRSV infection. Further research in these areas can provide a comprehensive understanding of viral spread within tissues, the dynamics of host-virus interactions, and how various host cell types contribute to the pathogenesis and immune response against PRRSV.

Furthermore, the functional roles of ncRNAs in PRRSV infection and replication should be explored. Additional investigations into the regulatory mechanisms of host ncRNAs in modulating viral replication, immune responses, and disease outcomes could lead to the development of novel therapeutic interventions and identification of potential biomarkers for diagnostic purposes.

Additionally, incorporating multi-omics approaches (such as transcriptomics, genomics, proteomics, and epigenomics) will provide a more comprehensive understanding of the complex molecular interplay between PRRSV and the host. The integration of these omics datasets could uncover crucial interactions, pathways, and networks involved in PRRSV infection and host responses that ultimately lead to more effective preventive and therapeutic strategies.

Lastly, efforts should continue to focus on sustainable pig farming practices that reduce reliance on antibiotics and mitigate environmental impacts. This includes integrating genetic information into breeding programs to select disease resistance traits, advancing precision farming technologies for early detection and intervention, and promoting biosecurity measures to minimize the risk of pathogen transmission.

In conclusion, future PRRSV studies should advance our understanding of the genetic and molecular mechanisms underlying host-virus interactions, develop targeted control strategies, and promote sustainable pig farming practices. Harnessing these insights will facilitate more effective prevention, management, and control of PRRSV that benefit swine health and productivity.

## 8. Conclusion

Overall, the findings of this review contribute to a comprehensive understanding of the genetic factors underlying the varied responses to PRRSV in different pig breeds to PRRSV infection. This knowledge can be used to formulate more effective control measures for PRRSV, such as designing breeding programs to select resistance traits and developing targeted vaccines. Additionally, exploring the potential of genetic-based interventions and further research into host-virus interactions at the molecular level holds promise for future developments in PRRSV control and prevention.

## Author contributions

YP: Writing – original draft, Writing – review & editing. CL: Writing – original draft. HL: Writing – review & editing. ZF: Writing – original draft, Writing – review & editing.
